# Human Endogenous Retroviruses as Pathogenic Factors in the Development of Schizophrenia

**DOI:** 10.3389/fpsyt.2015.00183

**Published:** 2016-01-11

**Authors:** Gorjan Slokar, Gregor Hasler

**Affiliations:** ^1^Psychiatric University Hospital, University of Bern, Bern, Switzerland

**Keywords:** schizophrenia, human endogenous retrovirus, HERV-W, pathogenesis, gene–environment interaction, infectious

## Abstract

Schizophrenia is a complex disorder, characterized by the interplay between genetic and environmental factors. Human endogenous retroviruses (HERVs), genetic elements that originated from infections by exogenous retroviruses millions of years ago, comprise ~8% of the human genome. Here, we provide a comprehensive review of accumulating evidence, detailing HERV aberrancies associated with schizophrenia. Studies examining the genome, transcriptome, and proteome of individuals with schizophrenia provide data that support the association of these viral elements with the disorder. Molecular differences can be found within the central nervous system and peripheral tissues. However, additional studies are needed to substantiate the reported link and to address several discrepancies among previous investigations. We further discuss potentially relevant pathogenic mechanisms to the development of schizophrenia.

## Introduction

The pathogenesis of schizophrenia is presently hypothesized to involve complex interactions between genetic and environmental factors (1). Occurrence of such interactions during critical phases of human neurodevelopment is thought to underlie disease initiation (2). Recent efforts have identified numerous candidate gene loci that confer increased susceptibility to this devastating disorder (3–5). Considering environmental influences, infectious agents have long been suspected to be involved in the etiology of schizophrenia (6), and accumulating evidence supports this theory. Being born during winter and spring (7) and living in relatively more urbanized areas (8) are both linked to an increased risk of exposure to infectious pathogens and to the development of schizophrenia. Prenatal maternal infections by *Toxoplasma gondii*, influenzavirus, and HSV-2 heighten the child’s risk of being diagnosed with the disorder at a later stage in life (9). Inflammatory markers associated with infection are elevated in peripheral blood (10) and cerebrospinal fluid (CSF) samples of subjects with schizophrenia (11), and occurrence of higher values of certain cytokines during childhood increases the probability of developing psychosis as a young adult (12). Moreover, several gene loci associated with schizophrenia are found in genomic regions that determine the response of the immune system to pathogens (4). However, the mechanism by which diverse infectious agents might be involved in the etiology of schizophrenia has not yet been identified. In this review, we summarize evidence regarding a possible missing link between infectious pathogens and schizophrenia: the so-called human endogenous retroviruses (HERVs).

Endogenous retroviruses (ERVs) are endogenous viral elements that are derived from past infections of germ cells by exogenous retroviruses. The first infection of a human ancestor by an exogenous retrovirus is estimated to have taken place around 100 million years ago (13). Many more integration events have occurred since, until possibly as recently as 100,000 years ago (14), which has resulted in HERVs comprising about 8% of the present human genome, distributed over several hundred thousand loci (15). Given the time scale of these integrations, currently, these elements are shared among different human populations, with only a few loci displaying insertional polymorphisms (presence/absence) (16, 17). Nearly all HERVs have accumulated mutations that prevent them from forming infectious virions or retrotransposing within the cell. Epigenetic silencing mechanisms further interfere with these processes, therefore, the inheritance of HERVs is limited to the classical Mendelian pattern. HERVs can be grouped into three classes, according to their similarities with existing exogenous retrovirus genera. Class I, Class II, and Class III contain *Gammaretrovirus*-like, *Betaretrovirus*-like, and *Spumavirus*-like HERVs. They can be further classified into “Families,” based on varying criteria such as sequence similarity or primer-binding sites. HERV classification has been extensively reviewed (18–20). HERVs exhibit the same basic gene structure as do exogenous retroviruses: 5′-LTR-*gag-pro-pol-env-*LTR-3′. The long terminal repeats (LTRs) contain promoter and enhancer sequences, necessary for transcription. *Gag, pro, pol*, and *env* encode the different proteins needed for the retroviral life cycle. However, most HERVs simply consist of a solitary LTR, missing the retroviral genes and the second LTR. Such HERVs are believed to have originated from homologous recombination of 5′ and 3′ LTRs. Several physiological functions of HERVs have started to be recognized in recent years. Recombination events involving HERVs have contributed substantially to the current structure of the human genome (21). LTRs of retroviral elements have been incorporated into the regulatory region of human genes to serve as promoters or enhancers (22). Apart from their significance for protein-coding genes, the role of HERVs in the evolution and regulation of non-coding RNAs is increasingly being recognized (23). In addition to their importance at the DNA level, HERVs themselves are also transcribed into RNA, depending on tissue type and the developmental stage (24, 25). The physiological significance of most of these transcripts has not yet been clearly established. Some transcripts are translated into proteins and perform essential functions in the host. The most well-studied instance is the function of *env-*derived proteins in the formation of the syncytiotrophoblast (26). HERV protein expression can also be observed in other tissues of healthy human individuals; however, the physiological role of these proteins has not yet been elucidated (27–29).

## HERVs and Schizophrenia

Human endogenous retrovirus have been implicated in various diseases, such as cancer (30), autoimmune diseases (31–33), and neuropsychiatric illnesses. In the following summary, we will focus on HERV-related findings in schizophrenia. Our summary is structured based on the molecular focus of each study (genetics, RNA expression, protein expression, and antibodies), and the different tissues investigated (brain, CSF, leukocytes, or plasma/serum).

### DNA Studies

DNA studies focusing on the role of HERVs in schizophrenia have tried to determine whether subjects with schizophrenia possess unknown HERV insertions/deletions or known polymorphisms correlate with the disease; an overview of the included studies has been presented in Table 1. In a study of three monozygotic twin pairs discordant for schizophrenia, a sequence designated schizophrenia-associated retrovirus 1 (SZRV-1) was amplified by polymerase chain reaction (PCR). Subsequent application of representational difference analysis yielded increased quantities of this sequence in all three affected twins (34). The observed sequence exhibited high similarity to the multiple sclerosis-associated retrovirus (MSRV). Three other sequences were further amplified, which were not increased in all the affected twins. When one of these sequences, termed SZRV-2, was used as a probe for Southern blot, the detection rate did not differ between eight subjects and their matched controls (35). Investigation of HERV-W *env* (MSRV subtype) copy number by real-time polymerase chain reaction (qPCR) in 59 subjects with schizophrenia revealed a significantly reduced copy number in the subjects compared to healthy controls (36).

**Table 1:** **Genetic studies on HERVs in schizophrenia**.

Study	Subjects	Target sequence	Main findings
Deb-Rinker et al. (34)	3 Monozygotic twin pairs discordant for schizophrenia	Retroviral *pol*	SZRV-1^a^ identified by representational difference analysis
Yolken et al. (37)	Schizophrenia	c-myc pseudogene	Pseudogene present in some affected subjects
	Healthy controls		
Deb-Rinker et al. (35)	8 Schizophrenia	SZRV-2	No differences detected
	21 Healthy controls		
Otowa et al. (38)	17 Schizophrenia	HERV-K115	HERV-K115 presence not increased. Tendency toward increased presence in subjects with younger onset (*p* = 0.057)
	181 Healthy controls		
Dickerson et al. (39)	229 Schizophrenia	HERV-K18 SNPs^b^	Haplotype associated with type 2 diabetes in SZ (*p* < 0.001, odds ratio = 9)
	136 Healthy controls		
Nyegaard et al. (40)	750 Schizophrenia	HERV-K18 SNPs	No association with type 2 diabetes. Tendency toward association with type 2 diabetes in younger schizophrenia subjects (*p* = 0.18)
	1214 Healthy controls		
Perron et al. (36)	59 Schizophrenia	HERV-W *env* (MSRV^c^)	Copy number of HERV-W *env* reduced in schizophrenia (*p* < 0.001) and bipolar disorder (*p* = 0.001)
	110 Bipolar disorder		
	105 Healthy controls		

*Here, we present a summary of genetic studies, examining genetic differences directly or indirectly relating to HERVs in individuals with schizophrenia and healthy controls. “Target sequence” denotes the sequences of the human genome used for PCR*.

*^a^Schizophrenia-associated retrovirus 1*.

*^b^Single nucleotide polymorphisms*.

*^c^Multiple sclerosis-associated retrovirus*.

A study investigating HERV-K115, a member of the HERV-K(HML-2) family displaying insertional polymorphism, found no association between HERV-K115 and schizophrenia (38). However, subjects with younger-onset schizophrenia exhibited a higher frequency of this retroviral element (38). On examining single nucleotide polymorphisms (SNPs) within the HERV-K18 locus in 229 schizophrenia subjects, one study found that a haplotype of two SNPs increased the risk of developing type 2 diabetes (odds ratio = 9) (39). A weaker association with type 2 diabetes was also found for several other SNPs of this locus (39). Another study could not confirm this association in a sample of 750 schizophrenia subjects, but observed a tendency toward an increased type 2 diabetes risk in subjects with younger-onset schizophrenia (40). They also found no association between any of the measured SNPs and an increased risk of developing schizophrenia.

Examination of the DNA of individuals with schizophrenia for the presence of processed pseudogenes of the c-myc transcription factor, which have not been detected in the genome of healthy individuals, revealed the presence of pseudogenes in some individuals with schizophrenia, while none were observed in healthy controls (37). Processed pseudogenes arise from the reverse transcription and integration of cellular messenger RNA into the nuclear DNA by retrotransposons.

### RNA Studies

An overview of the RNA studies has been presented in Table 2. In a study of brain tissue obtained from four schizophrenia subjects, application of primers specific for the retroviral *pol* region revealed that 45% of transcripts in schizophrenia subjects were homologous to HERV-W (MSRV) compared to 10% in controls (37). A subsequent study found the level of HERV-W transcripts to be significantly increased and that of ERV9 transcripts to be significantly decreased in the frontal cortex of five schizophrenia subjects (41). In a microarray study involving 35 schizophrenia subjects and 35 bipolar disorder subjects, no differences were found in the levels of HERV-W or ERV9 transcripts between subjects and controls; however, HERV-K10 transcripts were significantly increased in individuals with schizophrenia and bipolar disorder (42). Reverse transcription-polymerase chain reaction (RT-PCR) was also applied, targeting different HERV *env* sequences, and found no differences between samples obtained from healthy controls and individuals with schizophrenia (42). To evaluate the effect of psychiatric medication on HERV transcription, one study measured HERV RNA in different cell lines and in 18 brain samples obtained from subjects with schizophrenia and 20 brain samples obtained from subjects with bipolar disorder. Valproic acid (VPA) was found to induce increased RNA expression of certain HERVs *in vitro* with a particularly strong effect on the RNA expression of HERV-W and ERV9, but not on HERV-K (43). Tested antipsychotics had a minimal impact on HERV transcription (43). In the same study, ERV9 transcripts were significantly elevated in schizophrenia, and ERV9 and HERV-K (HML-2) transcripts were significantly elevated in bipolar disorder, compared to that observed in healthy controls independent of VPA treatment. HERV-W expression was not associated with schizophrenia or bipolar disorder. However, VPA-treated schizophrenia subjects exhibited significantly elevated HERV-W RNA levels, compared to those observed in untreated schizophrenia subjects. No such association could be found in VPA-treated bipolar disorder subjects.

**Table 2:** **Transcriptome studies on HERVs in schizophrenia**.

Study	Subject(s)	Specimen	Target sequence	Main findings
**Brain (postmortem)**				
Yolken et al. (37)	4 Schizophrenia	Frontal cortex	Retroviral *pol*	Increased HERV-W (MSRV) in schizophrenia. Increased HERV-K10 in bipolar disorder
	4 Bipolar disorder			
	6 Healthy controls			
Karlsson et al. (41)	5 Schizophrenia	Frontal cortex	Retroviral *pol*	Increased HERV-W (*p* < 0.0001). Significantly decreased ERV9 (*p* < 0.0001)
	6 Healthy controls			
Frank et al. (42)	35 Schizophrenia	Prefrontal cortex	Retroviral *pol*	Increased HERV-K10 in schizophrenia (*p* < 0.05) and bipolar disorder (*p* < 0.01)
	35 Bipolar disorder			
	35 Healthy controls			
	7 Schizophrenia		HERV-W *env*, HERV-FRD *env*, HERV-K *env*	No significant differences
	7 Healthy controls			
Diem et al. (43)	18 Schizophrenia	Prefrontal cortex	HERV-W *pol*, ERV9 *pol*, HERV-K(HML-2) *gag*	ERV9 increase in schizophrenia (*p* < 0.01). ERV9 and HERV-K(HML-2) increases in bipolar disorder (*p* < 0.01)
	20 Bipolar disorder			
	18 Healthy controls			Increases independent of medication
**CSF**				
Karlsson et al. (41)	35 Recent-onset schizophrenia or recent-onset schizoaffective disorder	Cell-free supernatant	Retroviral *pol*	Increased HERV-W, ERV9 and HERV-FRD expression (*p* < 0.001) 10/35, 1/20, 0/22, 0/30
	20 Chronic schizophrenia or chronic schizoaffective disorder			
	22 Non-inflammatory neurologic disease			
	30 Healthy controls			
**Blood**				
Karlsson et al. (44)	54 Recent-onset schizophrenia or recent-onset schizoaffective disorder or recent-onset schizophreniform disorder	Plasma	Retroviral *gag*	Increased HERV-W (*p* = 0.05) 9/54, 0/30
	46 Healthy controls			
Huang et al. (45)	58 Recent-onset schizophrenia	Leukocytes	Retroviral *pol*	Increased ERV9 (*p* < 0.01) 20/58, 0/38
	38 Healthy controls			
Yao et al. (46)	30 Recent-onset schizophrenia	PBMCs^a^	HERV-W *env, gag*	2.1-fold increase in *gag*, mainly from the element on 11q13.5 (*p* < 0.01). No *env* increase
	26 Healthy controls			
Huang et al. (47)	118 Recent-onset schizophrenia	Plasma	HERV-W *env*	Increased HERV-W *env* (*p* < 0.01) 42/118, 0/106
	106 Healthy controls			
Perron et al. (36)	45 Schizophrenia	PBMCs	HERV-W *env*	Increased transcripts in schizophrenia (*p* = 0.01) and bipolar disorder (*p* < 0.0001). Association remained after exclusion of VPA-treated subjects
	91 Bipolar disorder			
	73 Healthy controls			

*Here, we present a summary of studies examining differences in HERV transcription in individuals with schizophrenia in comparison to healthy controls. The table is divided into studies examining brain, CSF, and blood samples. “Specimen” denotes the exact origin/processing of samples. “Target sequence” denotes the sequence of the retroviral genome that was used for PCR*.

*^a^Peripheral blood monocular cells*.

In a study of CSF samples obtained from 35 subjects with recent-onset schizophrenia and 20 subjects with chronic schizophrenia, HERV RNA was detected in 10/35 subjects of the former group, 1/20 subjects of the latter group, and in none of the subjects of the non-inflammatory neurologic disease and healthy control group. The sequences identified belonged to the HERV-W, ERV9, and HERV-FRD families, with HERV-W constituting the majority of the sequences, and were reported to be particle-associated (41). Illness duration and symptom severity were not associated with the increase in HERV transcripts.

Based on their findings in the CSF, the presence of retroviral *gag* sequences was examined in the plasma samples of 54 recent-onset schizophrenia subjects. Particle-associated HERV-W RNA was found in 9/54 subjects and 2/46 controls (44). Five of the nine subjects whose plasma tested positive for particle-associated HERV-W RNA were also previously found to harbor retroviral RNA in their CSF, while only three subjects whose CSF tested negative for particle-associated HERV-W RNA were found to harbor retroviral RNA in their plasma. HERV-W-positive subjects showed a tendency toward increased acuity of psychotic symptoms (*p* = 0.06). Medication intake was not associated with HERV status. Another study detected HERV-W *env* RNA in the plasma of 42/118 recent-onset schizophrenia subjects, and in none of the controls (47).

ERV9 RNA was detected in the leukocytes of 20/58 subjects with recent-onset schizophrenia and 0/38 healthy controls (45). Another study tested peripheral blood monocular cells (PBMCs) of 30 first-episode schizophrenia subjects for the presence of HERV-W *env* and *gag* sequences. Multiple HERV-W elements were transcribed in both groups. *gag* transcripts were increased twofold in subjects with schizophrenia compared to those in healthy controls (46). A HERV-W element on 11q13.5 was identified as the main contributor. This element is inserted into the second intron of the putative gene *PTD015*. No differences were seen in *env* transcripts. *gag* transcripts exhibited a significant inverse relationship with illness duration. Symptom severity and medication intake was not associated with increased *gag* transcripts. The state of activation of PBMCs was further assessed by measuring the transcripts of two genes involved in metabolic pathways; however, no differences were found between schizophrenia subjects and healthy controls. Moreover, lymphocyte culture stimulation from three blood donors did not affect transcripts of the HERV-W *gag* gene detected in schizophrenia subjects. Exploration of the presence of HERV-W (MSRV) *env* sequences in PBMCs of 45 individuals with chronic schizophrenia revealed a significant increase in transcripts compared to that observed in controls and a significant decrease compared to that observed in bipolar disorder subjects (36). The association remained after exclusion of VPA-treated subjects. No association of HERV transcripts was found with illness duration or symptom severity. However, seropositivity against *T. gondii* was significantly increased in HERV-W-positive bipolar disorder and schizophrenia subjects, merged as a single group. Seropositivity against Herpes simplex virus 1/2 and Cytomegalovirus did not correlate with HERV-W detection.

### Protein Studies

An overview of the protein studies has been presented in Table 3. Reverse transcriptase activity was measured in the cerebellum of 12 schizophrenia subjects by the product-enhanced reverse transcriptase assay and found to be significantly increased compared to that in the controls (37). Significantly decreased levels of HERV-W GAG proteins were detected in the anterior cingulate gyrus and the hippocampus of 15 schizophrenia, 15 bipolar disorder, and 15 major depressive disorder subjects by immunohistochemistry (28). GAG proteins were expressed in neurons and astroglial cells. Analysis of GAG by western blotting yielded multiple bands, leading the researchers to conclude that multiple HERV-W elements contribute to this expression. The number of immune cells in brain tissue did not differ between subjects and controls. No association could be found between HERV-W GAG expression and serological status for *T. gondii*, Herpes simplex virus 1/2, and Cytomegalovirus.

**Table 3:** **HERV protein studies in schizophrenia**.

Study	Subjects	Specimen	Target	Main findings
**Brain (postmortem)**				
Yolken et al. (37)	12 Schizophrenia	Cerebellum	Reverse transcriptase	Increased reverse transcriptase activity in schizophrenia (*p* = 0.01) and non-psychotic depression (*p* < 0.05)
	12 Bipolar disorder			
	11 non-psychotic depression			
	12 Healthy controls			
Weis et al. (28)	15 Schizophrenia	Anterior cingulate gyrus, hippocampus	HERV-W GAG	Significant reduction of GAG in all subject groups (*p* ≤ 0.05)
	15 Bipolar disorder			
	15 Major depressive disorder			
	15 Healthy controls			
**Blood**				
Perron et al. (49)	49 Schizophrenia	Serum	HERV-W GAG	HERV-W GAG positive in 24/49 subjects, 2/49 controls (*p* < 0.001)
	49 Healthy controls			
	49 Schizophrenia		HERV-W ENV	HERV-W ENV in 23/49 subjects, 1/30 controls (*p* < 0.01)
	30 Healthy controls			
	35 Schizophrenia		CRP^a^	CRP correlation with ENV (*p* < 0.001) and GAG (*p* < 0.05) antigenemia
	49 Healthy controls			
Huang et al. (47)	118 Recent-onset schizophrenia	Serum	Reverse transcriptase	Increased reverse transcriptase activity (*p* < 0.05) 40/118, 3/106
	106 Healthy controls			

*Here, we present a summary of studies examining differences in protein expression relating to HERVs in schizophrenia subjects in comparison to healthy controls. The table is divided into studies examining brain and blood samples. “Specimen” denotes the exact origin/processing of the samples. “Target” denotes the retroviral protein assayed in the study*.

*^a^C-reactive protein*.

In serum samples, HERV-W GAG proteins were found in 24/49 individuals with schizophrenia and 2/49 controls, and HERV-W ENV in 23/49 individuals with schizophrenia and 1/30 controls (49). Detection of both proteins significantly correlated with C-reactive protein (CRP) levels. Medication intake was not associated with seropositivity for HERV-W proteins. Measurement of reverse transcriptase activity in the serum of recent-onset schizophrenia subjects by quantitative reverse transcription PCR (RT-qPCR) yielded activity of this enzyme in 40/118 schizophrenia subjects and 3/106 controls (47).

### Antibody Studies

Antibodies directed against a particular retroviral antigen can bind to homologous proteins of related retroviruses. Protein antigens of readily available retroviruses can, therefore, be used to detect antibodies directed against unidentified retroviruses. An overview of antibody studies has been presented in Table 4. HIV-1 western blot assay was applied to screen 38 schizophrenia subjects for the presence of retroviral antibodies. Antibodies were present in 21/38 subjects and 4/16 controls (50). Application of protein antigens of the betaretroviruses Mason-Pfizer monkey virus (MPMV), mouse mammary tumor virus (MMTV), simian retrovirus 5 (SRV-5), and *Gammaretrovirus* baboon endogenous virus (BaEV) revealed a significantly increased incidence of antibodies against MPMV, BaEV, and SRV-5 in 17 first-episode schizophrenia subjects (51). The largest differences were seen in antibodies against MPMV. The authors, therefore, assessed antibody levels against this species by immunoassay and immunoblot in 38 first-episode schizophrenia subjects and found that 11/38 subjects and 1/27 controls had antibodies above the set threshold (51). Follow-up of four seropositive subjects after 1 year revealed that two had reverted to a seronegative status and optical densities were diminished in the other two subjects. Using MPMV, the *Gammaretrovirus* murine leukemia virus (MuLV) and the *Lentivirus* feline immunodeficiency virus (FIV), significantly increased antibody levels against MPMV and MuLV were detected in 163 subjects with recent-onset psychosis, but not in 268 multi-episode schizophrenia subjects (52). These were directed against *gag*, *pol*, and *env* encoded proteins. Since the authors could not find any nucleic acids of the tested retroviruses in the subject sera and multiple regions of homology for both retroviruses were found within the human genome, they concluded that the detected antibodies likely arose from activation of homologous HERVs. Increased antibodies were not associated with symptom severity, illness duration, or medication intake. Antibodies against the ERV9 family were measured by *in vitro* expression of ERV9 POL proteins. Antibodies were present in the sera of some subjects, who also tested positive for ERV9 RNA, and absent in the sera of controls (45).

**Table 4:** **Retroviral antibodies in schizophrenia**.

Study	Subjects	Target	Main findings
Hart et al. (50)	38 Schizophrenia	HIV-1	Increased antibodies to HIV-1 antigens in SZ (*p* = 0.05) 21/38, 4/16
	16 Healthy controls		
Lillehoj et al. (51)	15 Recent-onset schizophrenia	MPMV^a^ GAG, BaEV^b^ ENV/GAG, MMTV^c^ GAG, SRV-5^d^ GAG	Increased incidence of antibodies to MPMV (*p* < 0.001), BaEV (*p* < 0.05), and SRV-5 (*p* < 0.05) antigens
	27 Healthy controls		
	38 Recent-onset schizophrenia	MPMV GAG	Increased antibody levels against MPMV (*p* < 0.01) 11/38, 1/27
	27 Healthy controls		
Huang et al. (45)	58 Recent-onset schizophrenia	ERV9 POL	Antibodies present in some ERV9 RNA-positive subjects
	38 Healthy controls		
Dickerson et al. (52)	163 Recent-onset psychosis	MuLV^e^, MPMV, FIV^f^	Increased antibodies to MuLV and MPMV in recent-onset psychosis (*p* < 0.001). No increase in multiple-episode schizophrenia
	268 Multiple-episode schizophrenia		
	235 Healthy controls		

*Here, we present a summary of studies examining the presence of retroviral antibodies in schizophrenia subjects and healthy controls. “Target” denotes the retroviruses/retroviral proteins used to assay the presence of antibodies*.

*^a^Mason-Pfizer monkey virus*.

*^b^Baboon endogenous virus*.

*^c^Mouse mammary tumor virus*.

*^d^Simian retrovirus 5*.

*^e^Murine leukemia virus*.

*^f^Feline immunodeficiency virus*.

### Potential Contributions of HERVs to the Pathogenesis of Schizophrenia

Different downstream effects of increased HERV activation may be involved in the pathogenesis of schizophrenia. Pro-inflammatory properties of HERV DNA, RNA, or proteins may be at the root of the immune aberrancies observed in individuals with schizophrenia. Normally, the immune response against viruses involves the detection of viral DNA and RNA within the cell. It has been proposed that HERV DNA and/or RNA might also activate such pathways (53). Such a mechanism is supported by a recent study, which details the exploitation of endogenous retroviral transcripts by the immune system, to induce a humoral immune response without the help of T-cells (54). More definite evidence has been obtained regarding the pro-inflammatory effects of HERV proteins. HERV-W ENV has been shown to induce an innate immune response via interaction with Toll-like receptor 4 (TLR4) and promote a TH1-like response, stimulating the release of IL-6, TNF-α, and IL-1β (55). The same protein also generates cytokine production in astroglial cells, induces death of oligodendrocytes, and may evoke superantigen-like effects (56, 57). *In vivo*, ENV and GAG antigenemia within serum significantly correlate with CRP levels (49). Multiple studies have repeatedly linked an increase in CRP, IL-6, TNF-α, and IL-1β to schizophrenia (10, 58), supporting inflammatory processes such as HERV-W-related ones being involved in the pathogenesis of this severe disorder.

Apart from its inflammatory effects, several other properties of HERV-W ENV have been proposed to be involved in the pathogenesis of schizophrenia. The human sodium-dependent neutral amino acid transporter type 1 (hASCT1) and hASCT2 have been established as the cellular receptors of HERV-W ENV (59). Both receptors play a role in the regulation of glutamatergic transmission in the brain (48), which has been found dysfunctional in schizophrenia. It has, therefore, been speculated that HERV-W ENV might contribute to schizophrenia by disturbing these pathways (44). Cell cultures infected by exogenous retroviruses, using an allelic receptor, do indeed exhibit impaired transport of the amino acids glutamine and alanine (60). Furthermore, a significant decrease in immunoreactivity of hASCT1 has been observed in neurons and astroglial cells of the cingulate cortex and hippocampus in individuals with schizophrenia, possibly reflecting occupation of the receptor by HERV-W ENV or receptor downregulation (48). Expression of HERV-W *env* in human glioma cells results in a two- to fourfold increase in expression of brain-derived neurotrophic factor (BDNF), neurotrophic tyrosine kinase receptor type 2 (NTRK2), and dopamine receptor D3 (DRD3) (47). BDNF aberrancies have been repeatedly associated with schizophrenia (61). In addition, the small conductance Ca^2+^-activated K^+^ channel protein 3 (SK3), which has been linked to schizophrenia as well, is also increased after expression of HERV-W *env* within neuroblastoma cells (62). Rats, receiving bilateral injections of recombinant HERV-W ENV protein into the ventral region of the hippocampus, exhibit several neurological and behavioral abnormalities associated with schizophrenia, including increased sensitivity to the psychostimulant MK-801, a common feature of animal models of schizophrenia, and a strong emergence of stereotypic behaviors, which are associated with some schizophrenia subtypes (63).

Aside from the effects of HERV products, HERVs might also contribute to schizophrenia via their regulatory effects on cellular genes. One HERV-W element was identified within the regulatory region of the GABA receptor B1 gene *GABBR1*, which is down-regulated in schizophrenia (64). A HERV-K(HML-2) element was confirmed to act as an enhancer for the schizophrenia-associated gene *PRODH* (65). It has been hypothesized that increased activity of one HERV element may lead to widespread epigenetic silencing of the whole respective HERV family, resulting in aberrant quantities of cellular proteins whose transcription is controlled by the individual HERV elements, such as GABA receptor B1.

In addition to their significance for protein-coding genes, the role of HERVs in the regulation of non-coding RNA potentially constitutes another pathogenic mechanism. HERVs are associated with a large number of long non-coding RNAs (lncRNAs), where they mainly overlap with transcriptional start sites (66). LncRNAs have a broad spectrum of biological activities, e.g., they can affect transcription, chromatin state, or splicing patterns, and are thought to be of particular importance in brain development (67). Evidence is emerging for an involvement of these molecules in disorders of the brain, including schizophrenia (68–70). It remains to be demonstrated whether any of the HERV transcripts observed in individuals with schizophrenia constitute functional non-coding RNA.

## Discussion

The most consistent evidence of an association between HERVs and schizophrenia comes from studies on HERV transcripts in blood samples of individuals with schizophrenia. Four studies found an increase in HERV-W transcripts, one study found an increase in ERV9 transcripts. Studies in postmortem brain tissue and CSF of schizophrenia subjects partly confirm these findings in blood. One study in CSF found increased HERV-W transcripts. Three studies in brain tissue found increased HERV-W, increased HERV-K10, and increased ERV9 transcripts. One possible reason for discrepancies regarding the associated HERV families in all of these studies may be related to technical differences and the high copy number of HERVs within the human genome. The HERV-W family is comprised of an estimated 250 elements and nearly all of these contain mutations, such as stop-codons, frame-shifts, or deletions (71). This sequence heterogeneity makes it very difficult to design primers for PCR, to amplify all members of a particular HERV family (24). Owing to the use of distinct primers in each study, different subsets of HERVs were likely amplified.

Four studies targeting retroviral proteins confirm findings of differential HERV activity at the protein level. Two studies found an increased reverse transcriptase activity, which is unspecific to any particular HERV family. One study found decreased HERV-W GAG, while another study found increased HERV-W GAG and ENV. Causes for decreased HERV-W GAG protein levels in the brain of individuals with schizophrenia are unclear, given the increases of this protein in the blood and at the RNA level. The authors themselves speculate that interfering mechanisms by non-coding HERV transcripts, disrupting the translation of coding transcripts, may yield an explanation (28).

Findings of HERV RNA and proteins in individuals with schizophrenia are further corroborated by the consistent detection of retroviral antibodies in this population. Four studies detected increased antibody levels to a *Lentivirus*, betaretroviruses, gammaretroviruses, and to the *Gammaretrovirus-*like ERV9. Considering evidence detailing HERV abnormalities associated with schizophrenia and the lack of evidence of an involvement of exogenous retroviruses in schizophrenia so far, it appears very likely that these antibodies are directed against HERVs. These antibody studies may additionally provide evidence for an involvement of the *Betaretrovirus-*like HERV-K in schizophrenia, since only one RNA study found an association with HERV-K. However, the detected antibodies against betaretroviruses may also simply be a result of cross-reactivity between antibodies against the *Gammaretrovirus*-like HERV-W or ERV9.

There is some evidence, indicating copy number variations of HERV-W in schizophrenia. One study using representational difference analysis found an increase in copy number, while another using qPCR found a decrease. Both studies suggest genomic alterations in schizophrenia subjects; however, more conclusive research is needed in this area.

There is preliminary evidence for the existence of c-myc pseudogenes in schizophrenia subjects. Although these have only been found in one study, they are of potential interest because this gene is mainly expressed during fetal development and may, thus, provide insights into the temporal development of environmental and genetic contributions to schizophrenia (37). These findings may, however, not only point to an involvement of HERVs but also point to the action of other retrotransposons, such as long interspersed nuclear elements (LINEs), also shown to be increasingly active in schizophrenia (37, 72).

Association of increased HERV activity with illness duration of schizophrenia subjects is debatable. Based on the initial findings in CSF, which were limited to recent-onset schizophrenia subjects, subsequent studies focused on subjects in the early symptomatic disease stages. Retroviral antibodies also seem to be exclusively found at disease onset. However, two studies analyzing the blood of chronic schizophrenia subjects and four post-mortem brain studies detected HERV abberrancies in later disease stages. Three studies found no association between illness duration and HERV expression/antibodies, while one study found an inverse relationship between illness duration and HERV RNA levels.

One *in vitro* study reported the influence of VPA on the transcription of HERV-W and ERV9. Two of six *in vivo* studies found an association between VPA intake and HERV-W expression, while the other four did not report any association. However, only one of these four studies separately analyzed different psychiatric drugs. The association of HERV-W with schizophrenia remained in one of the two positive studies after exclusion of subjects treated with VPA. It appears that VPA contributes, in part, to the HERV-related phenomena observed in schizophrenia. Future research will have to assess previous and current VPA intake to eliminate this confounding variable.

*In vitro* experiments have demonstrated that HERV expression can be induced by activation of monocytes (73). It has, therefore, been hypothesized that HERV-related findings in schizophrenia might simply be the result of increased immune cell activation or infiltration (42). However, the results of two studies in schizophrenia subjects do not support this premise, since immune cell activation in the blood and immune cell number in the brain did not differ in individuals with schizophrenia. Based on research detailing activation of HERVs after infection of immune (74) and neural cells (75–77), it has been speculated that transcriptional deregulation of HERVs in schizophrenia might be the result of an initial infectious event. HERV activation has been demonstrated in infections by schizophrenia-associated pathogens HSV-1 (76), *T. gondii* (75), and influenzavirus (76, 77). One study in schizophrenia subjects partially corroborates this hypothesis, since antibodies against *T. gondii* were associated with increased HERV-W transcripts, although no association was found with herpes viruses. Another smaller study in schizophrenia subjects could not find any association between infectious agents and HERV-W expression. It is important to note that HERV expression modulation in infection models is not limited to a single HERV family, such as HERV-W, but involves many different HERV families. It remains to be demonstrated whether the action of certain infectious agents may ultimately lead to similar changes in HERV expression over time, as have been observed in schizophrenia.

The association of HERV-W and schizophrenia is not highly specific. Bipolar disorder has also been associated with this HERV family, as has been reported in this review. Schizophrenia and bipolar disorder share approximately 60% of genetic risk factors (78) and exhibit similar brain structural abnormalities, such as reductions in total brain gray and white matter, and increased lateral ventricular volume (79). HERV-W has also been repeatedly linked with multiple sclerosis (80, 81). Schizophrenia and multiple sclerosis have been reported to share certain epidemiologic features, such as age of onset, season of birth, and geographic distribution (41). However, schizophrenia, bipolar disorder, and multiple sclerosis also exhibit differences regarding the molecular characteristics of HERV-W, which may be in part responsible for the observed differences in phenotype. As previously reported in one study, the DNA copy number of HERV-W is reduced in schizophrenia and bipolar disorder subjects. Conversely, examination of DNA copy number of HERV-W in multiple sclerosis subjects revealed increased copy numbers of this HERV family in the diseased subjects compared to healthy controls (82). Furthermore, the nucleotide sequences of examined HERV-W DNA and RNA slightly differ among all three disorders (36), possibly reflecting different subsets of HERV-W elements being activated.

Taken together, preliminary evidence suggests that differential activity of some HERV families is associated with schizophrenia in at least a subgroup of individuals with schizophrenia. The most frequently associated family is *Gammaretrovirus*-like HERV-W and to a lesser extent *Gammaretrovirus*-like ERV9 and *Betaretrovirus*-like HERV-K (HML-2). Limitations of the current evidence include the small number of studies, the high methodological heterogeneity, and the generally small study samples, including subjects with various diagnoses.

## Conclusion

Human endogenous retroviruses provide an intriguing new avenue of research into the constituent factors involved in schizophrenia. Establishment of a possible causal relationship may lead to the development of new treatment strategies. Such strategies may involve elimination of triggering environmental factors or direct inhibition of HERV RNA or proteins.

## Author Contributions

Both authors have reviewed the literature, structured the information, developed conclusions, and wrote the manuscript.

## Conflict of Interest Statement

The authors declare that the research was conducted in the absence of any commercial or financial relationships that could be construed as a potential conflict of interest.
